# Characteristics of deceased patients with CoVID-19 after the first peak of the epidemic in Fars province, Iran

**DOI:** 10.1080/20008686.2020.1781330

**Published:** 2020-06-17

**Authors:** Amir Emami, Fatemeh Javanmardi, Ali Akbari, Mohsen Moghadami, Hamid Bakhtiari, Farshad Falahati, Leila Hashemi Zadeh Fard Haghighi, Tahereh Rezaei

**Affiliations:** a Microbiology Department, Burn & Wound Healing Research Center, Shiraz University of Medical Sciences, Shiraz, Iran; b Department of Anesthesiology, School of Medicine, Shiraz University of Medical Sciences, Shiraz, Iran; c Non‐Communicable Diseases Research Center, Shiraz University of Medical Sciences, Shiraz, Iran; d Vice Chancellor for Health Affairs Center of Disease Control (CDC), Shiraz University of Medical Sciences, Shiraz, Iran; e Vice Chancellor for Treatment, Shiraz University of Medical Sciences, Shiraz, Iran

**Keywords:** Death, Covid-19, fatality rate, Iran

## Abstract

Emergence of a new coronavirus causes a serious concern whether this can be stopped at all. The ongoing coronavirus disease created a substantial variation in the fatality rate over the world. The current report brought an explore about the epidemiological characteristics of deceased patients and the fatality rate after the first peak in Fars province which is the fourth most populous and large province in Iran. Of the 3702 confirmed cases with coVID-19, 87 patients passed away and so the fatality rate estimated 2.35. Also, it was derived that male sex, old age and underlying diseases especially diabetes were common characteristics of these victims.

## Introduction

Corona virus disease 2019 (CoVID-19), a cause of respiratory and symptomatic/asymptomatic illness, has posed a global pandemic in 11 March 2020 and a medical emergency in the world. Individuals, populations, economics, and health care systems are bucking the burden of the disease besides the great dangers (mortality and morbidity) that has created for human society [1].

According to various reports; CoVID-19 usually begins with common symptoms including fever, cough, and shortness of breath. In severe cases, the infection may develop and causes pneumonia, organ failure, and death. CoVID-19 is identified as one of the most severe diseases in terms of rapid transmission and sudden fulminant complicate symptoms. In fact; this strain of coronavirus family is known as a highly contagious agent which is the main reason for the super spreading of the virus across the globe [2,3].

In accordance with the increasing number of infected cases during the pandemic, the case fatality rate has an ascending trend either of which some important reasons as following may justify this event: (1). prolong incubation time, (2). underlying diseases, 3. being a new disease, and so various diagnosis methods (high-resolution CT scan, molecular and serology methods for detection, and nucleic acid sequencing) [4].

Although, Chinese had first underestimated the case fatality rate and had given a false impression that CoVID-19 is like influenza with less death, but fortunately, based on previous experiences on the viral respiratory infections (such as influenza), and appropriate extensive personal health education (in community and centers), we could control the fatality rate by early specific readiness managements, in Iran and especially in the south, Fars province. From the initial days of onset of CoVID-19 in Fars (20 February 2020) until now, which first peak of the outbreak have been passes, (1 May 2020), the total number of confirmed cases were 3702 (among 43056 Tested cases) and the fatality rate was estimated 2.35 (87/3702). It is noteworthy that Fars is the fourth largest and the fourth most populous province in Iran (No: 4,851,274). The recent study is reporting the characteristics of death patients and related probable risk factors after experiencing the first peak of CoVID-19.

## Methods

The information about the characteristics of patients who died of COVID-19 were collected during the epidemic. All information was obtained and curated with data registry of Shiraz university of Medical Sciences. (Ethical code: IR.SUMS.REC.1399.022)

## Results

The overall age of 87 death patients was estimated 63.47 ± 22.04 years with the median of 67 years old (IQR: 52–82). According to the age category, it was revealed that most of the patients (25.30%) were more than 80 years. Among the fatal cases, 52 (59.8%) were males and the rest were females. Based on the history of underlying diseases in the dead’s, it was found only seven (6.36%) cases did not have any identified comorbidities, while diabetes was the most known common risk factor (27, 24.55%) which followed by cardiovascular diseases (23, 20.91%), hypertension (17, 15.45%), malignancy (10, 9.09%), kidney injury (8, 7.27%), COPD (4, 3.64%), asthma (2, 1.82%), CRF (3, 2.73%), vasculitis (1, 0.92%), lung disease (3, 2.73%), Zollinger–Ellison syndrome (1, 0.91%), hydrocephalus (1, 0.91%), and rheumatism (1, 0.91%), respectively. Further analysis showed that some cases had two risk factors simultaneously, so the sum of values is more than the number of patients. Based on the distribution of occupation most of the victims were house-wife (36, 41.1%), this is while 16 (18.4%) self-employee, 4 (4.6%) employee, and 2 (2.3%) were students, and none of them were health care providers or work in medical settings.

**Figure 1.**
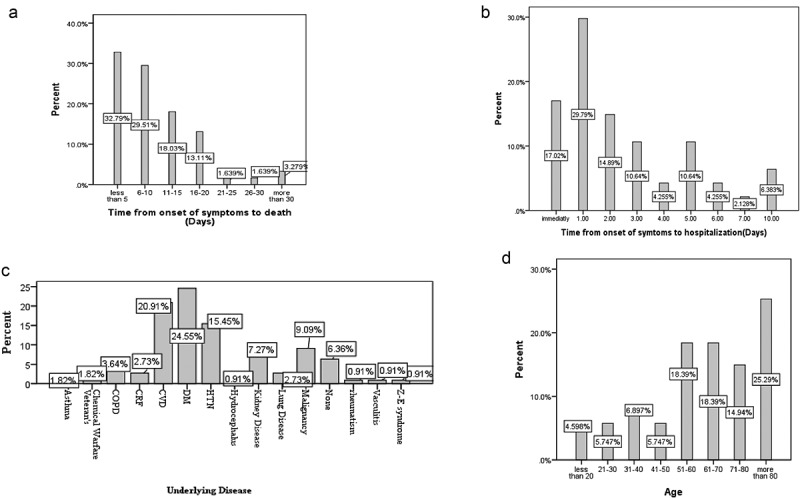
A. interval from onset of symptom to death of patients with confirmed COVID-19/B. interval from onset of symptom to hospitalization of death patients with confirmed COVID-19 C. Prevalence of underlying disease in death patients with confirmed CoVID-19/D. Age distribution of death patients with CoVID-19.

Due to the fact that timely referral to medical centers can play an important role in reducing the risk of death in the CoVID-19 infected people, we evaluated the interval from onset of symptoms to hospitalization either. Among patients, 17.02% were referred immediately after the appearance of symptoms. Figure 1 is shown these results.

## Discussion

This research aimed to report the characteristics of dead patients during the first peak of CoVID-19 pandemic in Fars, Iran. The results of this investigation support the fact that CoVID-19 fatality rate seems to be low in our province in comparison with other regions in Iran. This is while some disruptive factors like undetected cases and delayed reference may confound the current estimation. However, according to the results of the documentary, there are various reasons that justify this achievement. Some of these reasons are as following:

Extensive testing strategy and screening symptomatic and asymptomatic cases in the early phase of the outbreak play the first crucial role in improving health literacy and limited the transmission chain in Fars. Up to the end of first peak (1 May 2020) a total of 43056 tests were implemented, while 3702 cases were confirmed for CoVID-19. In fact, one of the main reasons for the successful management of infection was the average number of 1500 targeted tests per day in the province, which was high in comparison with other cities in Iran. This strategy was seen in the Republic of Korea and brought a successful implementation for this country to control this new virus either [5].

According to published reports, the case fatality rate in Italy was estimated 7.2% [6]. As it was clear in our results and another announcement, there is a strong association between age and CoVID-19 fatality rate, and this while Italy is known as an ageing population. About 37.6% of Italy’s population is 70 years and older [6,7]. An interesting and controversial result was seen either in the fatality rate and population age across New York City and its boroughs. Despite the low rate of older adults (age ≥65yeras) in the Bronx, but a high number of deaths related to CoVID-19 made a concerning issue. Some factors like poverty and low level of education may involve in this rate [8]. Although the association between mortality of CoVID-19 and comorbidities is approved in different reports but the notable point is the different pattern in underlying diseases. In current results, diabetes was identified as the common illness in the history of our studied patients, while other studies report hypertension and cardiovascular as the most prevalent underlying diseases [9,10].

The second possible explanation of health policy in controlling the transmission chain was screening the history movements in infected cases by use of Global Positioning System. Evaluating financial transactions of credit cards and movements map by cellular phone are all causative agents relating to low mortality rate due to CoVID-19 in Fars. Similar strategies were performed in Korea [5].

Overall, informing people in the community especially the high-risk groups (elderly, pregnant, patients with comorbidities), providing the medical supplies, increasing laboratory tests capacity, performing social distance, banning public gathering, and announcing quarantine were panic management strategies which implemented in Fars province and could moderate the death cases.

## Conclusion

According to the current analysis, the fatality rate estimated 2.35 during the first peak in Fars, one of the largest and most populous provinces in Iran. Also, it was derived that male sex, an underlying disease especially diabetes were common characteristics in deceased patients related to coVID-19.
